# Effect of Flavonols of *Aronia melanocarpa* Fruits on Morphofunctional State of Immunocompetent Organs of Rats under Cyclophosphamide-Induced Immunosuppression

**DOI:** 10.3390/biom14050578

**Published:** 2024-05-14

**Authors:** Kseniya Bushmeleva, Alexandra Vyshtakalyuk, Dmitriy Terenzhev, Timur Belov, Evgeniy Nikitin, Vladimir Zobov

**Affiliations:** A.E. Arbuzov Institute of Organic and Physical Chemistry, Kazan Scientific Center, Russian Academy of Sciences, Arbuzov Str. 8, Kazan 420088, Russia; alex.vysh@mail.ru (A.V.); dmitriy.terenzhev@mail.ru (D.T.); belofftimur@mail.ru (T.B.); berkutru@mail.ru (E.N.); vz30608@mail.ru (V.Z.)

**Keywords:** flavonols, berry extract, *Aronia melanocarpa*, histological analysis, cyclophosphamide, immunomodulatory effect

## Abstract

*Aronia melanocarpa* berries contain many compounds with potential benefits for human health. The food flavonoids quercetin and rutin, found in significant amounts in the fruits of *A. melanocarpa*, are known to have favourable effects on animal and human organisms. However, data on the effect of flavonols isolated from black chokeberry on immune functions during immunosuppression are not available in the literature. Thus, the aim of this study was to evaluate the effect of flavonol fraction isolated from *A. melanocarpa* fruits, in comparison with pure quercetin and rutin substances, on the dysfunctional state of rat thymus and spleen in immunodeficiency. The study was performed on Wistar rats. The animals were orally administered solutions of the investigated substances for 7 days: water, a mixture of quercetin and rutin and flavonol fraction of *A. melanocarpa*. For induction of immunosuppression, the animals were injected once intraperitoneally with cyclophosphamide. Substance administration was then continued for another 7 days. The results showed that under the influence of flavonols, there was a decrease in cyclophosphamide-mediated reaction of lipid peroxidation enhancement and stimulation of proliferation of lymphocytes of thymus and spleen in rats. At that, the effect of the flavonol fraction of aronia was more pronounced.

## 1. Introduction

The vertebrate immune system is a sophisticated defence mechanism against severe disturbances of homeostasis caused by pathogens, injuries, external pollutants and infections [1]. The thymus and spleen, as immune organs, are the main regulatory loci of immune function, with their development and maturity directly affecting the level of immunity [2,3,4]. The thymus is the primary lymphoid organ within which developing T-cells (thymocytes) proliferate and differentiate into a functional subpopulation of T-cells. The thymus tightly controls the antigenic specificity of naive T-cells entering the systemic bloodstream to limit reactions with autoantigens [5]. The thymus is an important immune organ responsible for T-cell generation and involved in adaptive immunity [6,7]. The spleen is both a site of T- and B-cell colonisation and immune response, with the potential to synthesise biologically active molecules [2,8]. The spleen has a crucial function in lymphocyte recirculation, B-cell differentiation and serving as a reservoir of memory B-cells [9]. Consequently, indicators of thymus and spleen condition are capable of reflecting the immune status of the organism [8].

The cytostatic drug cyclophosphamide (CP), a widely used clinical immunosuppressive antitumor drug used in the treatment of numerous cancers, is known to cause damage to healthy cells and provoke oxidative damage to the thymus, spleen, kidney, liver and other tissues [10,11,12,13,14]. CP is used for investigating the immunomodulatory effects of biologically active compounds because it has some immunosuppressive potential [15].

Flavonoids, which are a large group of plant-derived polyphenolic compounds and are mainly found in vegetables, fruits and medicinal plants, have an important function in free radical detoxification. Quercetin and rutin were reported to exhibit anti-inflammatory, antioxidant, antibacterial, radical scavenging, antiviral, immunoprotective, and immunomodulatory properties and were used for the treatment of cardiovascular diseases and obesity, both having therapeutic activity for oxidative stress-induced diseases [16,17]. 

The plant flavonoids quercetin and rutin (quercetin-3-O-rutinoside), which have a variety of health benefits [18], are abundant in the fruits of *Aronia melanocarpa*, a temperate plant that bears fruit well in temperate climates. There are literature data supporting the ability of *Aronia melanocarpa* fruit extract to inhibit the development of metastases and enhance the antimetastatic effect of CP [19].

The present study used a model of immunodeficiency in animals induced by CP to evaluate the immunomodulatory effect of the flavonol fraction of chokeberry. The function of flavonols in the regulation of immune organ activity is still largely unknown. Therefore, in order to fill the gap in knowledge about flavonols, particularly those isolated from *A. melanocarpa*, in the regulation of immunological functions, the present study aimed to evaluate the effect on the dysfunctional state of the thymus and spleen following exposure to anti-cancer drug cyclophosphamide.

## 2. Materials and Methods

### 2.1. Sampling

Aronia berries of the Black Pearl variety were collected and characterised as part of our previous study [20]. The mature Aronia berries were gathered in the Shujskie Yagodi farm (Ivanovo region). The berries had mass average of 1.1–1.3 g. Berries were randomly collected from ten bushes, in the amount of 0.5 kg per plant. At the time of berries harvest the chokeberry bush was 6 years old. The berries were washed with distilled water to remove dust and contaminants, dried with cotton cloth, and stored in the freezer at −35 °C for further research.

#### 2.1.1. Extract Preparation

The flavonol fraction of *Aronia melanocarpa* fruit extract was prepared and characterised according to the methods described in [21]. The frozen and crushed of *Aronia melanocarpa* berries were extracted using the maceration method with 90% ethanol solution and 1.3% potassium dihydrophosphate to create a buffer system with pH = 5.5 for 3 h at 45 °C in a water bath. The obtained homogenate was centrifuged and concentrated on a rotary evaporator over the water bath. The method of column chromatography was used to purify and recover the flavonol fraction [22]. Darco G-60, a modified carbon chromatographic sorbent, with particle size distribution from 0.7 to 1.0 mm was used as the stationary phase.

The sorbent material was placed in a glass column, and washed with the elute selected by TLC method, consisting of the solvent system of ethanol: ethyl acetate (1:9). Then the investigated extract was added, and seven eluted fractions were collected. The fraction obtained was dried until the elute evaporated. For the studies, the dried flavonol fraction was dissolved in 1.3-propylene glycol to produce 1% of the solution.

#### 2.1.2. Chemicals and Reagents

Quercetin standards, purity ≥95.0%; quercetin-3-O-rutinoside (rutin), purity ≥94.0%; were purchased from PhytoLab GmbH & Co. KG, Germany (Merck KGaA, Darmstadt, Germany).

Following the described method for the preparation of flavonols, an enriched extract with a flavonol content of ≥88.3% was obtained from chokeberry berries. The major compounds quercetin and rutin with purity ≥91.1% and ≥91.2%, respectively, were isolated from this extract.

### 2.2. Ex Vivo Experiments

All procedures and animal manipulations were approved by the Ethical Committee of Kazan (Volga Region) Federal University (protocol No. 4 dated 18.05.2017). The studies were conducted on adult male Wistar laboratory rats weighing 300–400 g with induced reduction of immune functions (immunosuppression) due to administration of CP cytostatic drug. The animals were kept in accordance with References [23,24] in standard conditions in a vivarium with 12-h daylight and free access to food and water. The animals were fed with complete feed made according to specification (protein, 22%; fibre, 4% max.; fat, 5% max.; ash, 9% max.; humidity, 13.5% max.; caloric value, 295 kcal/100 g).

During the experiment, rats prophylactically received orally water (control group or group C, *n* = 6), the flavonol fraction of black chokeberry fruit extract at a dose of 50 mg/kg per dry weight (group P) and a solution of quercetin and rutin in concentrations equivalent to the flavonol fraction of the extract (group Q) for 7 days. The ratio of purified flavonols—quercetin and rutin in the mixture of standards was 1.3:1. The dose of the mixture was 4.33 mg/kg. The doses of the flavonol fraction of the extracts were from the literature [25,26,27] and from the results of our previous studies, which showed a positive effect of chokeberry fruit extracts at a dose of 50 mg/kg [28]. The volume of administered solutions and extracts to rats was 0.1 mL per 100 g of body weight. Drinking water in the control group was administered in equivalent amounts to create similar exposure conditions as in the experimental groups. 

For induction of immunodeficiency, rats on the 8th day of the experiment were once intraperitoneally administered CP at a dose of 25 mg/kg and further continued to be administered orally with the investigated substances during the next 7 days of the experiment.

Blood was collected lifetime from the tail vein—the animal was placed in a holder, and the tip of the tail was disinfected with 70% ethanol and cut off with sharp scissors. Blood was drawn from the wound in the volume of 0.5–1 mL into vacuum tubes with anticoagulant ethylenediaminetetraacetate (EDTA). 

The state of immunodeficiency after CP administration was confirmed by the data of peripheral blood analysis, according to which on the next day after CP administration the number of leukocytes decreased almost twice and there was also observed a change in the ratio of subpopulations and functional state of immune cells [20].

#### 2.2.1. Histological Analysis of Thymus and Spleen

To assess the state of immunocompetent organs of thymus and spleen in rats, histological examination of these organs was performed. At the end of the experiment the animals were euthanised by chloral hydrate injection and thymus and spleen were removed for studies and prepared to estimate mass index and examine structure. Organ samples were taken from each animal and placed in histological cassettes. The thymus was taken whole for study, and 5 × 10 × 5 mm pieces were cut from the spleen. 

The samples were subjected to rehydration which was performed on a Sakura Tissue-Tek^®^ VIP™ 5 Jr automatic enclosed vacuum histological processor (Sakura, Nagano, Japan) according to the protocol. After rehydration, the specimens were embedded into Histomix paraffin medium using the MtPoint ESD-2800 (MtPoint, Saint Petersburg, Russia) paraffin filling station. The specimens were embedded into plastic moulds, with the histological cassette serving as a base for the future paraffin block.

From the obtained cassettes, 5 µm thick sections were made on a Sakura Accu-Cut SRM 200 rotary microtome (Sakura, Nagano, Japan), which were placed in a bath with distilled water heated to 37 °C to straighten them. Then, the most suitable slice was extracted from the tape and placed on a glass slide treated with a special BioVitrum adhesive medium.

Haematoxylin–eosin staining was used in this work, as it allows to reveal almost all cells and many non-cellular structures. Due to this staining, it is possible to describe the general morphology of the tissue, to see all the changes in it. Harris haematoxylin and aqueous eosin solution were used for staining. The stained sections were enclosed in synthetic mounting medium Vitrogel (BioVitrum, Saint Petersburg, Russia) and placed under cover-glass.

Morphometric analysis and microphotography were performed using a Nikon Eclipse E200 (Nikon, Tokyo, Japan) direct light microscope with a Nikon digital camera and NIS-Elements Imaging Software 3 (Nikon, Kawasaki, Japan). 

#### 2.2.2. Thymus Tissue Analysis

On histological sections of thymus, stained with haematoxylin and eosin, the area of structural elements of thymus tissue was determined, expressing them in square millimetres. The percentage of the thymic medulla (TM) area relative to the observed area of the thymus lobule was calculated according to the Formula (1):

(1) $$\mathrm{Proportion}\;\mathrm{of}\;\mathrm{thymic}\;\mathrm{medulla},\;\%=\frac{\mathrm{TM}\;\mathrm{area}}{\mathrm{Lobule}\;\mathrm{area}}\times 100\%,$$

Percentage of thymic cortex (TC) area relative to the visualised area of the thymus lobule was calculated according to the Formula (2):

(2) $$\mathrm{Proportion}\;\mathrm{of}\;\mathrm{thymic}\;\mathrm{cortex},\;\%=\frac{TC\;\mathrm{area}}{\mathrm{Lobule}\;\mathrm{area}}\times 100\%,$$

The ratio of the medulla lobules to the cortex of rat thymus was also calculated (3):

(3) $$\mathrm{Relative}\;\mathrm{indices}\;\mathrm{of}\;\mathrm{thymus},\;\%=\frac{\mathrm{TM}\;\mathrm{area}}{\mathrm{TC}\;\mathrm{area}}\times 100\%\;$$

#### 2.2.3. Spleen Tissue Analysis

On spleen sections stained with haematoxylin and eosin, the area of structural elements of the spleen tissue was determined and expressed in square millimetres. We calculated relative indices (in %) of structural components of rat spleen, such as white pulp (WP) area, separately for lymphoid follicle (LF) (4) and marginal zone (MZ) (5), red pulp (RP) area (6) using the following formulas:

(4) $$\mathrm{LF}\;\mathrm{WP}\;\mathrm{area},\;\%=\frac{\mathrm{LF}\;\mathrm{WP}\;\mathrm{area}}{\mathrm{Observed}\;\mathrm{area}\;}\times 100\%,$$

(5) $$\mathrm{MZ}\;\mathrm{WP}\;\mathrm{area},\;\%\;=\frac{\mathrm{WP}\;\mathrm{area}-\mathrm{LF}\;\mathrm{WP}\;\mathrm{area}\;}{\mathrm{Observed}\;\mathrm{area}\;}\times 100\%,$$

(6) $$\mathrm{RP}\;\mathrm{area},\;\%=\frac{\mathrm{RP}\mathrm{area}}{\mathrm{Observed}\;\mathrm{area}\;}\times 100\%,$$

The ratio of white pulp to red pulp of the spleen was also calculated using the Formula (7):

(7) $$\mathrm{Relative}\;\mathrm{indices}\;\mathrm{of}\;\mathrm{spleen},\;\%=\frac{\mathrm{WP}\;\mathrm{area}}{\mathrm{RP}\;\mathrm{area}}\times 100\%,$$

#### 2.2.4. Effect of the Aronia Flavonol Fraction on Spleen and Thymus Indices

To study the immunoregulatory effects of the flavonol fraction of aronia, the indices, or mass ratios, of spleen and thymus were calculated as follows (Formulas (8) and (9)):

(8) $$\mathrm{Spleen}\;\mathrm{index},\;\%=\frac{\mathrm{Spleen}\;\mathrm{weight}\;\left(g\right)\;}{\mathrm{Animal}\;\mathrm{weight}\;\left(g\right)}\times 100\%,$$

(9) $$\mathrm{Thymus}\;\mathrm{index},\;\%=\frac{\mathrm{Thymus}\;\mathrm{weight}\;\left(g\right)\;}{\mathrm{Animal}\;\mathrm{weight}\;\left(g\right)}\times 100\%,$$

#### 2.2.5. Determination of the Level of Lipid Peroxidation in the Blood of Rats

The content of malondialdehyde (MDA) in rat red blood cells was determined using the thiobarbituric acid reactive substance assay as described by Buege and Aust [29] with a slight modification of the method. Saline-washed erythrocytes of 0.1 g were homogenised in 0.15 mol L^−1^ KCl at a ratio of 1:9 using a glass homogeniser. One volume of erythrocytes was thoroughly mixed with two volumes of a mother liquor solution of 15% wt./vol. trichloroacetic acid, 0.375% wt./vol. thiobarbituric acid and 0.25 mol L^−1^ hydrochloric acid. The solution was heated for 15 min in a boiling water bath. After cooling the precipitate was removed by centrifugation at 1000× *g* for 10 min. The absorbance of the clear supernatant was determined at 535 nm and the MDA concentration was calculated using 1.56 × 10^5^ mol^−1^ cm^−1^ as the molar absorption coefficient. MDA results were expressed as µmol per litre of blood with initial moisture content.

### 2.3. Statistical Analysis

The findings were processed using Microsoft Excel 2016 and OriginPro 9.5 (OriginLab Co., Northampton, MA, USA). Samples were compared using the non-parametric Kruskal–Wallis test. In cases of the normal distribution, the comparison of samplings was performed by Student’s *t* test. In cases of non-normal distribution, the comparison was performed by the Mann–Whitney test. SPSS 23.0 statistical software (Chicago, IL, USA) was used to analyse the data. The results were presented as mean values ± standard deviation of the mean. The *p* level <0.05 was considered statistically significant.

## 3. Results

### 3.1. Effect of Flavonols on Immune Organ Mass Indices

High doses of CP exposure activate the loss of body weight, thymus and spleen, which inhibits the development and function of T-cells, B-lymphocytes (B-cells) and natural killer cells [30,31]. Consequently, it has been widely used to create animal models with immunosuppression [32,33,34]. To observe the effect of flavonol compounds on the immunosuppressive state of animals induced by CP, we determined the body weight of rats and the mass of extracted central and peripheral organs of the immune system—thymus and spleen, respectively. 

During the analysis of animal body weight, no negative effect of administration of a single dose of cyclophosphamide on the dynamics of body weight change was detected, because animals continued to gain weight physiologically during the experiment (Table 1). Administration of rutin–quercetin solution (group Q), as well as flavonol fraction of aronia (group P), to the animals during the experiment resulted in slower body weight gain.

**Figure 1:**
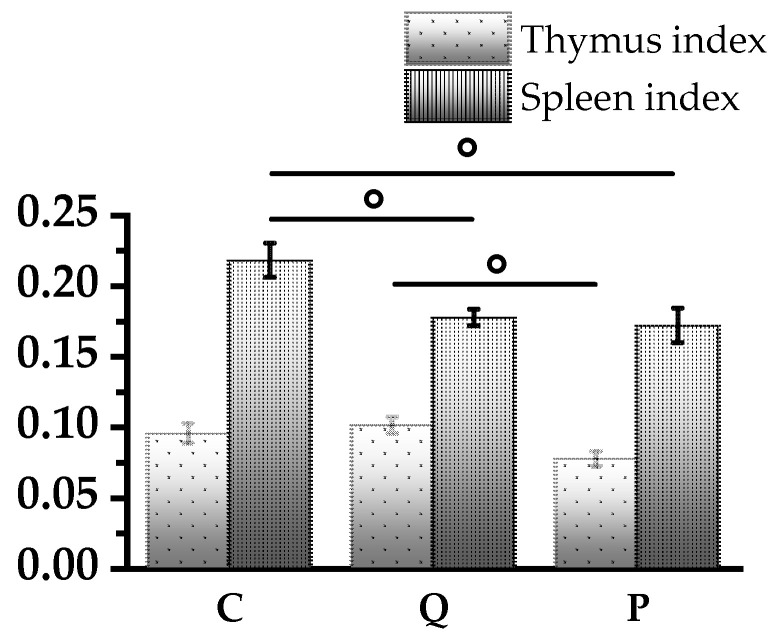
Histogram of the spleen and thymus index of each group on day 21 of the experiment. Results are presented as mean values ± standard deviation (*n* = 6). ◦ (*p* < 0.05)—Statistically significant differences between groups (*t* test).

In this study, we calculated the indices of the spleen and thymus index of the above groups, defined as the ratio of the organ weight to the animal weight (Figure 1). The analysis of thymus indices of the experimental groups did not reveal significant differences with the control group, however, in the Q group the index was higher by 19% in comparison with the P group (*p* < 0.05) and by 13.1% in comparison with controls. 

The spleen index was reduced relative to the control group by 19% in group Q (*p* < 0.01) and by 21% in group P (*p* < 0.05). At the same time, there was no significant difference in the values of spleen index between the experimental groups Q and P, which indicates an equal effect of aronia flavonols and quercetin–rutin mixture.

### 3.2. Evaluation of Morphological Changes in Rat Thymus and Spleen Tissues Using Light Microscopy

After exposure to CP and further therapy with flavonols, the morphological state of the rat thymus and spleen was evaluated on the 21st day of the experiment. This study allowed us to evaluate the effect of the tested substances on the state of central (thymus) and peripheral (spleen) organs of the immune system against the background of CP-induced rat immunosuppression.

It is known that the main processes occurring in the thymus are proliferation, differentiation, death and migration of thymocytes. Their reproduction is mainly carried out in the subcapsular zone of the cortex, from where maturing thymocytes move to the medulla, acquiring phenotypic features characteristic of a subpopulation of peripheral T-cells in the process of contact with stromal cells. The study of thymus microanatomy allows us to judge the state of these processes and, consequently, the reactivity of the immune system both at the organ level and as a whole.

**Figure 2:**
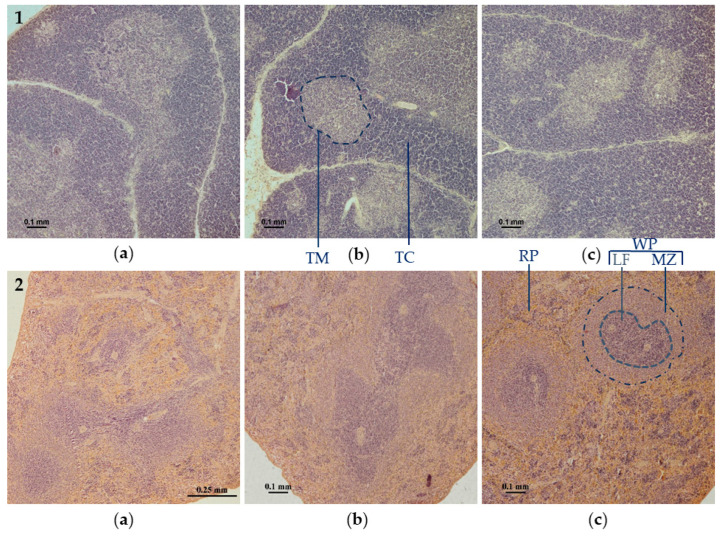
Representative data of morphological changes of thymus (**1**) and spleen (**2**) tissues of rats (magnification 10×, staining with haematoxylin and eosin). (**a**) Control group rat (CP); (**b**) Q group rat (CP + rutin–quercetin mixture 4.33 mg/kg); (**c**) P group rat (CP + aronia flavonol fraction 50 mg/kg). The spleen is divided into two main zones: red pulp (RP) and white pulp (WP), including marginal zone (MZ), and lymphoid follicle (LF)—objects are bounded by a dotted line. The thymus is divided into thymic medulla (TM) thymic cortex (TC).

Therapy with flavonol compounds after administration of CP at a dose of 25 mg/mg promoted positive changes in thymus structure. The experimental groups had thicker thymic cortex and a smaller proportion of cortex compared to the control group, indicating a higher immune activity of the tissue. The thymus lobule was differentiated clearly, borders were clear (Figure 2(1)).

At the same time, in the control group, the thymic cortex was thinner, and the proportion of the medullary zone was significantly larger. The lobule was smaller and less clear interlobular borders were detected. The erasure of borders can be explained by thymocyte migration and their mass decay, as well as by suppression of proliferative activity. Thus, therapy with both flavonol fraction of aronia and individual flavonols quercetin and rutin markedly led to a significant improvement in the morphological structure of the thymus, indicating its higher functional activity. 

Similarly, by analysing the morphology of the spleen in the groups of animals after flavonol treatment, we could observe that the size of spleen lymph nodes was similar, the border between white pulp and red pulp was clear, and the border area was delineated (Figure 2(2b,c)). For the control group, the border between white pulp and spleen lymph node was not clear, which corresponded to the histological characteristics of reduced immune activity of the spleen. The changes indicative of the immunosuppressive state of the spleen in the control group included a small volume of white pulp and width of the marginal zone, sparse lymph nodes and disappearance of germinal centres in them, disorganisation of periarterial lymphatic sheaths (PALSs). In addition, in the control group, compacted cytoplasm of cells, irregular angular contours were observed (Figure 2(2a)).

One of the determinable morphological parameters of the thymus is the ratio of structural components, namely, the cortex and medulla. These data allow us to better understand the structure of immune structures, and to link functional characteristics and they are convenient to use when comparing the effect of different pharmacological substances on the thymus.

**Figure 3:**
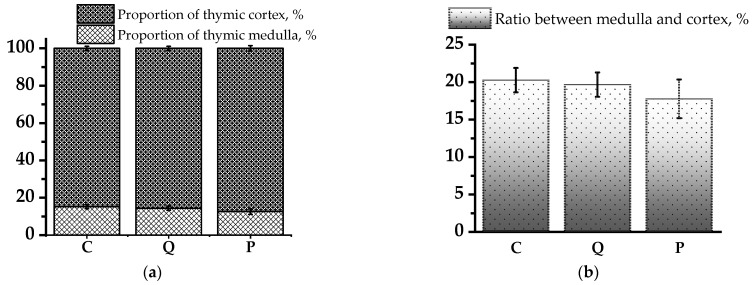
Ratio of structural components of rat thymus. (**a**) Proportion of thymic cortex and medulla (%); (**b**) Ratio between the medulla and cortex of thymus (%).

Figure 3 shows the ratio of structural components of the thymus of the control group of rats subjected to cyclophosphamide administration only, as well as the corresponding indices in the groups of rats treated with flavonol compounds. There was a trend towards an increase in the cortical zone of the thymus in the groups receiving the flavonol fraction of aronia, compared to the control, which is a numerical confirmation of the activation of the proliferative activity of thymus tissue shown above in the description of histological preparations (Figure 3). In the group of rats receiving the quercetin and rutin solution, the proportion of thymic cortex (TC) was higher by 0.84%, and in the group of rats receiving the flavonol fraction of aronia, higher by 2.63% compared to the control group. The obtained results confirm the higher activity of the flavonol fraction of aronia compared to pure quercetin and rutin substances.

**Figure 4:**
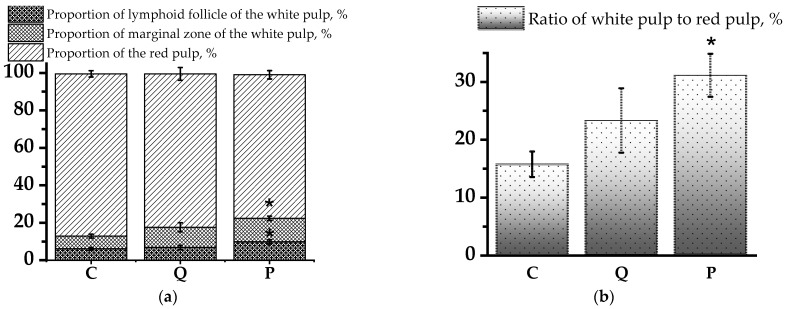
Ratio of structural components of rat spleen. (**a**) Proportion of lymphoid follicle and marginal zone of the white pulp and the red pulp of spleen (%); (**b**) Ratio of the white pulp to the red pulp (%).Results are presented as mean values; error bars indicate the standard deviation. * (*p* < 0.05)—Statistically significant differences compared to the control group (*t* test).

Histological analysis of spleen sections revealed a significant increase in the proportion of lymphoid follicles and marginal zone of the white pulp (WP) in absolute number by 1.6 and 1.8 times, respectively, in the group of rats receiving the flavonol fraction of aronia compared to the control group (Figure 4a) (*p* < 0.05). After therapy with the flavonol fraction of aronia, the area of the white pulp of the spleen was increased relative to the red pulp (RP) (*p* < 0.05). Thus, under the action of aronia flavonols, a significant improvement in the structural organisation of the spleen was observed, indicating a decrease in the signs of CP-induced immunosuppression detected in the analysis of histological preparations of the spleen in the control group (Figure 4b). It should be noted that the quercetin–rutin mixture had a less pronounced effect and did not lead to a reliable improvement of the structural and functional state of the spleen.

### 3.3. Effect of Flavonols on Lipid Peroxidation Parameters

MDA, a quantitative indicator of the lipid peroxidation (LP) level in rats, was investigated to determine the extent of antioxidant properties of the tested substances in an ex vivo model (Table 2). In the control group, the intensification of LP processes on day 21 increased by 18.5% compared to the initial level (*p* < 0.05). Similarly, but slightly less pronounced in the group receiving quercetin and rutin solution, on day 21 the level of LP increased by 10.1% relative to day 1 (*p* < 0.05). In the group of rats receiving flavonol fraction of aronia, intensification of LP processes was not detected—MDA level on day 21 even decreased by 5.3% and was lower than the control group by 33.5% (*p* < 0.001).

## 4. Discussion

There are findings that supporting the body’s defence system through food and/or herbal treatments can benefit the gut microbiota, as well as lead to a reduction in inflammation processes, increased resistance to viral infections and alleviation of nutritional imbalances [35]. Consequently, it is crucial to develop immunomodulators that are both safe and effective for immunosuppression or immunodeficiency states. Numerous studies have shown that natural products derived from plants can strengthen the immune system [36,37,38,39].

Flavonols, found in significant quantities in the fruit of chokeberry, have many biologically active functions, contributing to the body’s health status [40,41,42]. To determine the function of the flavonol fraction of chokeberry in the regulation of immunity, we created a model of immunosuppression in rats by induction of CP, and to restore immune dysfunction we performed therapy with the flavonol fraction of chokeberry fruit extract, using a mixture of quercetin and rutin flavonols as comparison substances.

Data show that the mass of immune organs increases with increasing proliferation, differentiation and activation of immune cells and vice versa [43,44]. Therefore, the change in the relative mass of immune organs has an important informative value in assessing the state of immune function. It was found that in the groups receiving a mixture of rutin and quercetin, the thymus index was greater than in the control, which may indicate higher activity of lymphoid tissue [45]. The higher thymus index in the experimental groups compared to the control may be associated with a slowdown in the processes of age-related thymic involution under the influence of the studied substances, which contributes to a longer preservation of the organ’s activity and improvement of the immune functions of the organism.

Spleen index indices in the experimental groups Q and P were significantly reduced compared to the control group. However, this may not necessarily be related to the suppression of the immune function of the spleen, since this organ also contains a large amount of erythrocyte mass and fulfils the function of blood depot, participating in the regulation of blood flow. An increase or decrease in the mass of the spleen can be caused by an increase or decrease in blood filling of the organ. Therefore, the real state of the immunocompetent tissue of the spleen can be assessed by the data obtained during histological studies.

Positive dynamics of body weight gain were observed throughout the experiment, but administration of quercetin–rutin mixture as well as flavonol fraction of chokeberry contributed to slower body weight gain. Previously, Lim et al. presented data on the effect of aronia flavonoids on body weight loss due to changes in leptin levels [46]. Qin and Anderson [47] found that an ethanol-water extract of *A. melanocarpa* reduced the weight gain and risk factors associated with insulin resistance by modulating the adipogenic signalling cascade. Some results show beneficial effects of *A. melanocarpa* along with improvements in body weight, liver functions, lipid profiles and antioxidant capacity suggesting the potential therapeutic efficacy of *A. melanocarpa* [48].

Analysis of histological preparations showed that oral administration of flavonol fraction of aronia promoted a significant stimulation of lymphocyte proliferation in the white pulp of the spleen, and also resulted in a tendency to increase the thymic cortex. The effect of the rutin–quercetin mixture on structural indices of the thymus and spleen was similar but less pronounced. These results are consistent with the data on the effect of *A. melanocarpa* extract leading to a decrease in histological damage caused by CP in the delayed period [49]. Also, these results are in agreement with the data on the immunomodulatory effect of flavonols of *A. melanocarpa*, shown in our previous work [20]. First of all, the obtained effects may be associated with the chemical composition of plant complexes, namely, with a high content of phenolic compounds, polyphenols, which, in turn, is the reason for one of the highest levels of antioxidant and anti-inflammatory activity of chokeberry fruits among other berries [50].

Our study showed that in the group that took orally flavonol fraction of *A. melanocarpa*, the level of malondialdehyde in the blood showed a tendency to decrease compared to baseline values, in contrast to the control and the group that received quercetin–rutin mixture, in which MDA increased by the end of the experiment by 18.5% and 10.15%, respectively. The obtained results indicate more pronounced antioxidant properties and ability to inhibit lipid peroxidation processes in the flavonol fraction of *A. melanocarpa* compared to the known standard antioxidants quercetin and rutin. The revealed properties of flavonols of *A. melanocarpa* can undoubtedly be due to the presence of antioxidant molecules other than quercetin and rutin, which significantly enhance antioxidant properties. Although the mechanism of the immunomodulatory effect of *A. melanocarpa* flavonols is not fully studied, it should be assumed that one of the possible mechanisms of the revealed properties may be the high antioxidant activity of this group of substances.

## 5. Conclusions

The study showed that flavonol compounds isolated from *A. melanocarpa* fruits, namely quercetin and rutin in synergy with other compounds of flavonoid nature, can significantly attenuate CP-induced immune stress and oxidative damage by protecting central and peripheral organs of the immune system, as demonstrated in the thymus and spleen.

Thus, the extract of the flavonol fraction of chokeberry can be used as a dietary supplement to combat immunosuppression and to reduce the side effects of cyclophosphamide therapy against tumours.

## Figures and Tables

**Table 1:** Effect of flavonols on body weight gain in rats (g) exposed to cyclophosphamide.

	Group C ^1^	Group Q ^2^	Group P ^3^
Day 1	362.5 ± 9.1	322.5 ± 7.2 °	326.3 ± 14.3
Day 21	390.8 ± 8.4 *	344.5 ± 6.7 *°	352.5 ± 14.4

^1^—group of rats orally received water—control group or group C; ^2^—group of rats received the solution of quercetin and rutin—group Q; ^3^—group of rats received the flavonol fraction of *A. melanocarpa* fruit extract—group P; * (*p* < 0.05)—Statistically significant differences compared to day 1 of the experiment; ° (*p* < 0.05)—Statistically significant differences compared to the control group (*t* test).

**Table 2:** MDA level (µmol/L) of peripheral blood of rats.

	Group C ^1^	Group Q ^2^	Group P ^3^
Day 1	31.66 ± 1.16	29.75 ± 0.77	26.35 ± 0.55 *
Day 21	37.53 ± 2.69 °	32.77 ± 0.81 °	24.95 ± 0.85 **

^1^—group of rats orally received water—control group or group C; ^2^—group of rats received the solution of quercetin and rutin—group Q; ^3^—group of rats received the flavonol fraction of *A. melanocarpa* fruit extract—group P; results are presented as mean values ± standard deviation (*n* = 6); * (*p* < 0.05) ** (*p* < 0.001)—Statistically significant differences compared to the control group; ° (*p* < 0.05)—Statistically significant differences compared to day 1 of the experiment (Mann–Whitney test).

## Data Availability

Dataset available on request from the authors.
